# Development of a diagnostic compatible BCG vaccine against Bovine tuberculosis

**DOI:** 10.1038/s41598-019-54108-y

**Published:** 2019-11-28

**Authors:** Aneesh Chandran, Kerstin Williams, Tom Mendum, Graham Stewart, Simon Clark, Sirine Zadi, Neil McLeod, Ann Williams, Bernardo Villarreal-Ramos, Martin Vordermeier, Veerasamy Maroudam, Aravind Prasad, Neeraj Bharti, Ruma Banerjee, Sunitha Manjari Kasibhatla, Johnjoe McFadden

**Affiliations:** 1 School of Biosciences and Medicine, Faculty of Health and Medical Sciences, University of Surrey, Guildford, GU2 7XH UK; 2 Animal and Plant Health Agency, Addlestone, Surrey UK; 3 Public Health England, Porton Down, Salisbury, UK; 4 Translational Research Platform for Veterinary Biologicals, Chennai, India; 5 HPC-Medical and Bioinformatics Applications Group, Centre for Development of Advanced Computing, Innovation Park, Panchavati, Pashan, Pune, 411008 Maharashtra India

**Keywords:** Infectious-disease diagnostics, Live attenuated vaccines

## Abstract

Bovine tuberculosis (BTB) caused by *Mycobacterium bovis* remains a major problem in both the developed and developing countries. Control of BTB in the UK is carried out by test and slaughter of infected animals, based primarily on the tuberculin skin test (PPD). Vaccination with the attenuated strain of the *M. bovis* pathogen, BCG, is not used to control bovine tuberculosis in cattle at present, due to its variable efficacy and because it interferes with the PPD test. Diagnostic tests capable of Differentiating Infected from Vaccinated Animals (DIVA) have been developed that detect immune responses to *M. bovis* antigens absent in BCG; but these are too expensive and insufficiently sensitive to be used for BTB control worldwide. To address these problems we aimed to generate a synergistic vaccine and diagnostic approach that would permit the vaccination of cattle without interfering with the conventional PPD-based surveillance. The approach was to widen the pool of *M. bovis* antigens that could be used as DIVA targets, by identifying antigenic proteins that could be deleted from BCG without affecting the persistence and protective efficacy of the vaccine in cattle. Using transposon mutagenesis we identified genes that were essential and those that were non-essential for persistence in bovine lymph nodes. We then inactivated selected immunogenic, but non-essential genes in BCG Danish to create a diagnostic-compatible triple knock-out ΔBCG TK strain. The protective efficacy of the ΔBCG TK was tested in guinea pigs experimentally infected with *M. bovis* by aerosol and found to be equivalent to wild-type BCG. A complementary diagnostic skin test was developed with the antigenic proteins encoded by the deleted genes which did not cross-react in vaccinated or in uninfected guinea pigs. This study demonstrates the functionality of a new and improved BCG strain which retains its protective efficacy but is diagnostically compatible with a novel DIVA skin test that could be implemented in control programmes.

## Introduction

Bovine TB (BTB) is one of the most complex animal health problems that the world’s cattle industry faces today and which causes significant economic losses^1,2^. BTB also causes zoonotic TB, prompting, in 2017, the first road map to combat zoonotic TB to be rolled out^3–5^. The active surveillance and control of BTB in most parts of the world depends on animal screening by tuberculin skin testing and the removal of test-positive animals by culling^6^. The tuberculin skin test is performed by injection of small quantities of *M. bovis* purified protein derivative (PPD-B), which is a crude and complex antigenic mixture that provokes a visible immune reaction on the skin^7^. To mitigate cross-reactivity with environmental mycobacteria and to optimise specificity, in the so-called comparative tuberculin test, PPD-B is sometimes supplemented by injection of PPD derived from the related *Mycobacterium avium* (PPD-A). Many countries also use the so-called single intradermal test relying only on the injection of PPD-B, a test format optimised for sensitivity, also often used as international trade test. Tuberculin skin testing is compulsory in many countries. Yet control by test-and-cull is expensive and societally and/or economically unacceptable in many countries. It also fails to achieve TB eradication in some epidemiological circumstances^8^.

Vaccination is nearly always the most cost-effective means to control infectious disease. The BCG vaccine was developed for control of human tuberculosis by Albert Calmette and Camille Guerin by repeated sub-culturing of the bovine TB bacillus, *Mycobacterium bovis* until it lost virulence for guinea pigs yet protected them from challenge with live virulent TB bacilli. The BCG vaccine was first used in humans in 1921 and has since become the most widely used vaccine for humans^9^. Experimental approaches to developing an improved vaccine against TB have included the use of attenuated mycobacteria, subunit vaccines, and DNA vaccines^10,11^. Several live attenuated mycobacterial vaccines have shown promising efficacy against *M. tuberculosis* challenge in animal models^11–15^ and have progressed to clinical trials^16,17^. There is also evidence that deletion of genes from BCG can result in unaltered or even superior protective efficacy compared to the corresponding parental BCG^18^. BCG is also effective in protecting cattle against TB and has been shown to be capable of reducing the number, duration and severity of herd breakdowns^19–22^. However, it is not used to control BTB since BCG shares many antigens with *M. bovis*^23,24^ resulting in the failure of the PPD skin test to distinguish between a BCG-vaccinated and BTB-infected cow^25^.

The discovery that the development of BCG from *M. bovis* involved the concomitant deletion of large regions of the BCG chromosome (the RD regions)^26,27^ stimulated the search for antigens that have been lost in the BCG genome and may therefore be used in a novel test capable of Differentiating Infected from Vaccinated Animals (DIVA). Antigens such as ESAT-6 and CFP10, both encoded on the RD1 region have been used as DIVA antigens^28,29^ in humans and cattle blood tests (interferon-gamma release assays, IGRA)^28–30^. However, a drawback of the IGRA tests is that they are laboratory-based requiring blood samples to be taken from infected animals, transported to the laboratory within a tight timeline and under temperature-controlled transport conditions. The test result must then be reported back to the field. These requirements make the IGRA DIVA tests expensive and inappropriate for developing countries with limited resources and technological infrastructure^31^. In contrast, a DIVA skin test would overcome these limitations^28^.

Recently a defined diagnostic skin test was developed based on a cocktail of DIVA antigens, ESAT-6, CFP-10 and Rv3615c, that allow the differential diagnosis of *M. bovis*-infected from BCG-vaccinated animals^29,32,33^. The specificity of this DIVA test is high but its sensitivity is less than the SIT test^32,34,35^. Although adding additional *M. bovis* antigens to the cocktail would likely increase its sensitivity, this is not a viable option since no other potential DIVA antigens have been identified within the RD regions of BCG; and adding non-DIVA *M. bovis* antigens to the cocktail will likely decrease the specificity of the test.

Our aim was to develop a new BCG vaccine that is compatible with a sensitive and affordable DIVA skin test. The approach we used is based on the observation that at least three *M. bovis* antigens (ESAT-6, CFP-10 and Rv3615c) are dispensable for the protective efficacy of BCG. This led us to hypothesize that the programme of accidental deletion of antigens from *M. bovis* initiated by Calmette and Guerin to develop the BCG vaccine, could be continued by rational design. We therefore initiated a project to construct a novel strain of BCG, ΔBCG, deleted in additional antigens dispensable for protection. Vaccination with ΔBCG would thereby provide us with the opportunity of adding additional antigens, deleted from the ΔBCG genome, to the current DIVA cocktail that might provide an enhanced sensitivity skin test reagent capable of detecting BTB infection in ΔBCG inoculated animals.

It is well established that persistence of BCG in the host is crucial for its protective efficacy^36^. In a previous paper we describe the development of a high throughput transposon mutagenesis approach that was used to identify BCG genes that influence the ability of the vaccine to persist in the bovine host^37^. In this paper we describe the use of that dataset to identify BCG genes encoding antigens that do not impair the ability of BCG to persist in the bovine host and are therefore candidate genes that could be deleted. We go on to describe the construction of a ΔBCG antigen-depleted strain that it is safe and induces protective immunity comparable to wild-type BCG against *M. bovis* infection in guinea pigs. In parallel we also describe the development of a skin test diagnostic based on detection of antigens deleted from the ΔBCG strain. This study provides a novel approach to development of rationally-designed live vaccines.

## Results

The starting point for our experiments was the identification of genes that influence the survival of BCG in the bovine lymph node. The details of these experiments are fully described elsewhere^37^, with the method based on the original BCG lymph node challenge model^38^. Briefly, a BCG Danish transposon (Tn) library was constructed and inoculated into the prescapular lymph nodes of three calves. The library was recovered from lymph nodes after 3 weeks and the input and output library pools were compared by Tn-seq and TRANSIT’s Resampling method analysis^39^ to identify genes that, when inactivated by the transposon, influenced persistence in bovine lymph nodes^40^. Genes that did not influence persistence were deemed dispensable and were therefore considered candidate for deletion in the engineered ΔBCG strain. Genes in the list of dispensable loci that encoded antigens were identified by cross-checking against a list of 500 proteins whose immunoreactivity in TB-infected cows has been already characterized^41,42^ to identify dispensable antigenic proteins.

**Figure 1:**
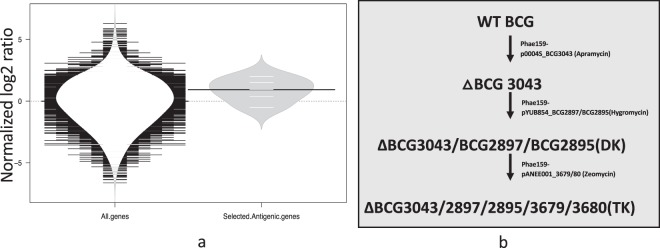
(**a**) Bean plot of fold changes during *in vivo* passage in cattle for selected gene groups and (**b**) Schematic representation of creating △BCG TK. (**a**) Black lines show the medians; white lines represent individual antigenic genes; polygons represent the estimated density of the data. The grey regions is a density plot of the data’s distribution. Values are normalised to the median value of the ‘All gene’ group. The plots were created with BoxPlotR (Spitzer, Wildenhain *et al*. 2014) (**b**) △BCG TK was created by sequential gene knock out using specialized Transduction method (Bardov *et al*. 2002). The Phagemid used was Phae159 and the cosmids used were p0004S, pYUB854 and pANEE001 to create △BCG 3043, ΔBCG3043/BCG2897/BCG2895 (Double knock out) and ΔBCG3043/2897/2895/3679/3680 (Triple knock out) respectively.

Five genes encoding antigens were identified as Tn mutants in the library whose fold changes during *in vivo* passage in cattle was between 0.5 to 2-fold (Fig. 1a), indicating that they were not essential for persistence in the bovine lymph condition, and so could be eliminated from the genome via three genome deletions without compromising the ability to persist in cattle. The genes selected were BCG3043, BCG2897, BCG2895, BCG3679 and BCG3680 which are orthologs of *M.tb* genes Rv3020c, MPT70, MPT83, Rv3615c and Rv3616c, respectively. Rv3020c is a member of the ESX family of virulence factors known to induce a potent cellular immune responses^43^. Rv3615c [Esx-1 substrate protein C (EspC)], encoded outside of RD1, is an antigen that is already part of the DIVA skin test prototyope described by Whelan *et al*.^33^. The MPB70 and MPB83 antigens were reported to be highly specific and sensitive for the detection of *M. bovis* infection in animals without any cross-reaction with *Mycobacterium* spp. found in the environment^44–46^. Lastly, Rv3616c is a CD8 antigen in mice with characteristics of the Esx family of bacterial proteins^47^. It is also strongly recognised by T cells from infected cattle^48^. We therefore chose to delete these genes from BCG Danish.

### Construction of modified ΔBCG TK vaccine

All 5 antigen genes were removed using specialized transduction method^49^ by three sequential deletion steps each using vectors with different antibiotic cassettes for the selection of mutants at each stage (Fig. 1b) to make a ΔBCG TK mutant. PCR analysis of the genetic constructs^50^ created to make the deletion mutants were all of the expected size (Sup. Fig. 2).

### Growth analysis of ΔBCG TK mutants in standard growth medium and in bovine macrophages

**Figure 2:**
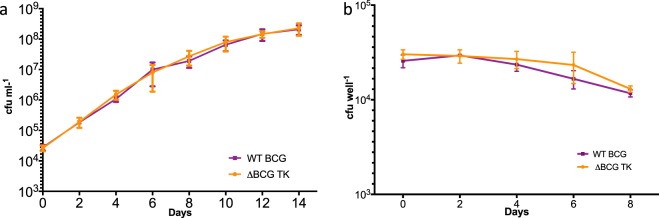
Competitive survival of selected mutants *in vitro* and *ex vivo* conditions. Competitive *in vitro* survival of selected mutants in (**a**) media and (**b**) bovine macrophages. Approximately equal number of WT BCG Danish and BCG knockout were mixed and used to inoculate media, infect PBMC derived bovine macrophages. Inoculants and recovered BCG were enumerated on selective media. Error bars represent standard errors.

To confirm that the deletion of the genes did not have any growth defect, we first tested the *in vitro* growth kinetics of the mutant strain compared to WT BCG in a competition assay. When co-cultured with wild type BCG in 7H9 media the TK mutant did not show any loss of fitness when compared to WT (Fig. 2a). We next sought to determine whether the deletion of BCG antigenic genes influenced the survival of BCG in bovine macrophages. PBMC-derived bovine macrophages were infected with the mixture (1:1) of WT BCG and the ΔBCG TK mutant. The ΔBCG triple mutant survived in the macrophages as well as WT BCG (Fig. 2b). This confirms that the removal of antigens does not alter the *in vitro* or *ex vivo* growth characteristics of the ΔBCG TK strain.

### Protective efficacy of ΔBCG TK in guinea Pigs

**Figure 3:**
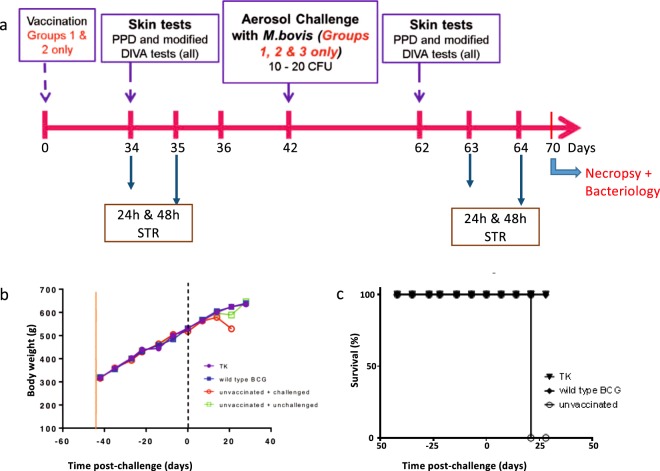
Comparative analysis of body weight and survival ability of ΔBCG TK vaccinated Guinea pigs after *M. bovis* challenge. (**a**) Schematic representation of study schedule (**b**) Group mean body weight profiles recorded for each group of guinea pigs during the vaccination and challenge phase of the study. Solid red vertical bar on x-axis indicates the time point of vaccination and dashed-black vertical bar on x-axis indicates time point of challenge with *M. bovis*. (**c**) Survival data of unvaccinated and vaccinated (WT BCG, ΔBCG TK) guinea pigs up on *M. bovis* infection.

The aerosol-infection guinea pig model of human TB and bovine TB is commonly used as a screening tool to assess the protective efficacy of vaccines^51,52^. *M. bovis* challenge of guinea pigs has also proven useful to test the potency of vaccines against bovine TB^53^. Dunkin Hartley guinea pigs were randomly divided into four groups. Group 1 and Group 2 were immunised subcutaneously on the nape with ΔBCG TK (5 × 10^4^ cfu) and the wild-type BCG respectively. Group 3 and Group 4 were unvaccinated controls. Protective immunity was assessed as the ability to reduce disease progression following challenge at 42 days post-vaccination (Fig. 3a) with virulent *M. bovis* (10–20 cfu delivered by aerosol). Disease progression was assessed by weight loss, a sensitive indicator of TB in the guinea pig model. Disease burden was quantified by measurement of viable *M. bovis* in lungs and spleens.

The uninfected controls (Group 4) together with all animals immunized with either ΔBCG TK or wild-type BCG gained weight normally after challenge (Fig. 3b). Indeed, there were no significant differences in weight gain between these two groups. After 21 days post-infection, however, non-immunized, infected animals (Group 3) exhibited a substantial weight loss and were euthanised at a pre-defined humane end-point; whilst the weight of vaccinated guinea pigs continued to increase steadily (Fig. 3b).

Although this study was not powered to measure survival, the notable difference between disease progression in vaccinated and unvaccinated animals permitted an analysis of survival (based upon time to humane end-point). The Kaplan Meier plot (Fig. 3c) shows that the vaccinated and challenged guinea pigs all survived until the end of the 4-week post-infection observation period, whereas all of the unvaccinated-challenged group had been euthanised by this time. In addition, none of the animals vaccinated with either wild-type or ΔBCG strains showed any pathological signs of clinical disease thus confirming the protective efficacies of the strain.

**Figure 4:**
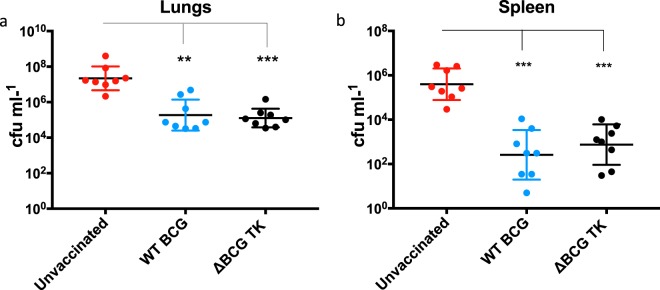
Protective efficacy of ΔBCG TK vaccination in Guinea pigs upon *M. bovis* challenge. (**a**) The bacterial load in the lungs of WT BCG (P = 0.0021) and ΔBCG TK (P = 0.0006) vaccinated guinea pigs is significantly low compared to the with the unvaccinated control group. (**b**) The bacterial load in spleen of WT BCG (P = 0.0015) and ΔBCG TK (P = 0.0006) vaccinated guinea pigs is significantly low compared to the unvaccinated control group. Values for each individual are shown and the horizontal bar denotes the mean for each group. *P = ≤ 0.05, P = ≤ 0.01, ***P = ≤ 0.001.

To assess the capacity of the recombinant vaccine ΔBCG TK to restrict the growth of *M. bovis* in tissues of challenged guinea pigs, the number of viable bacteria (colony forming units, cfu) in the lung, the primary site of infection, and spleen, a major site of bacterial dissemination was quantified at necropsy. The cfu data from lungs (Fig. 4a) and spleens (Fig. 4b) of challenged animals showed statistically significantly lower cfu burden in the lungs (p < 0.001) and spleens (p = 0.0004) of animals vaccinated with either vaccine compared to unvaccinated controls. The reductions in bacterial burden in either organ imparted by vaccination with wild-type BCG and ΔBCG TK was indistinguishable (Fig. 4). Together with the data demonstrating prevention of disease progression, these data on reduced bacterial burden in lungs and spleens following vaccination with either vaccine demonstrates that the deletion of the target genes from ΔBCG TK has not reduced its protective efficacy.

### Skin test immune response against extended DIVA antigens in guinea pigs

To test whether the antigens deleted from ΔBCG TK could induce skin test responses in *M. bovis* infected guinea pigs, but not in vaccinated animals prior to infection, orthologs of the genes deleted from ΔBCG TK: Rv3020c (BCG3043), MPB70 (BCG2897), MPB83 (BCG2895), Rv3615c were prepared as three different fusion proteins (ESAT-6-CFP-10, MPB70-MPB83, Rv3615c-Rv3020c). These were tested alone, or in combination, as synthetic antigen cocktails.

**Figure 5:**
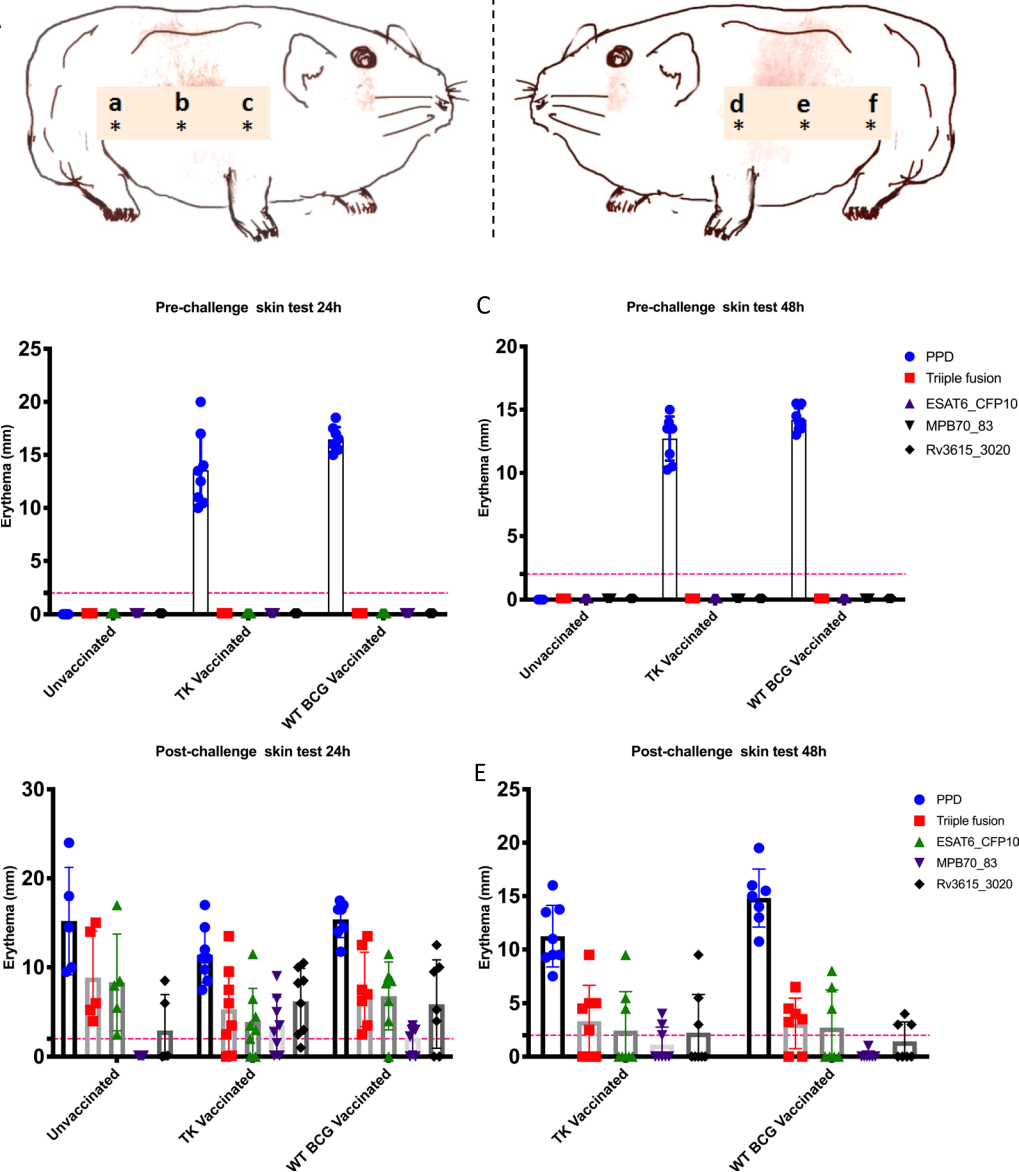
Pre and post-challenge skin test reaction in ΔBCG TK vaccinated guinea pigs. (**A**) Diagram to show the injection layout on animal. At least 2.5 cm between each inoculation of antigen preparations were injected on the same flank in a Latin Square arrangement^54^. The site of injections on both sides of guinea pig’s trunk was marked “a, b, c” and “d, e, f”. (**B,C**) Pre-challenge skin test: The group mean size of the diameter of erythema at 24 (**B**) and at 48 hours (**C**) after injection. (**D,E**) Post-challenge skin test: the mean size of the diameter of erythema at 24 (**D**) and 48 (**E**) hours after injection. The red line denotes the minimum skin test response (STR) threshold (2 mm) for DIVA antigens to consider it as positive. The x-axis denotes the vaccine group. The error bars indicate standard deviation for each vaccine group.

Groups of guinea pigs were vaccinated as above with either WT BCG, or ΔBCG TK, or left unvaccinated controls, and subsequently challenged with *M. bovis*. Skin tests were performed on all animals post-vaccination to determine specificities, and also performed again post-infection to determine the sensitivities of the test reagents. The following antigen preparations were injected in a Latin Square arrangement^54^ in the sites shown in Fig. 5A: PPD-B, fusion proteins of ESAT6-CFP10, MPB70 and MPB83, Rv3615c and Rv3020c. A cocktail of all of these three fusion proteins (Triple antigen cocktail) was also tested. The site of injections on both sides of guinea pig’s trunk was marked “a, b, c” and “d, e, f (Fig. 5A) and the skin-testing injection regimes were showed in Sup. Table 2.

The groups of animals vaccinated with WT BCG or ΔBCG TK gave no skin reactions with any of the DIVA antigen cocktails either 24 h or 48 h post vaccination before challenge with *M. bovis*. As expected, injection of the standard PPD-B skin test reagent gave rise to reactions in both groups of vaccinated guinea pigs, Unvaccinated animals did not respond to either the DIVA cocktails or to PPD-B (Fig. 5B,C).

Following *M. bovis* challenge of these animals, both vaccine groups, as well as the unvaccinated control group, showed consistently positive responses to PPD-B with no significant difference in response between the vaccinated and unvaccinated animals (Fig. 5D,E). Challenged animals also reacted to the DIVA antigen cocktails, particularly against the triple fusion protein cocktail injection (MPB70-MPB83, ESAT-6-CFP10 and Rv3615-Rv3020) at the 24 h and 48 h measuring time point (Fig. 5D,E). The response to the MPB70/83 DIVA antigen was however not consistently observed in all animals. Unvaccinated animals showed response to PPD-B and the antigen cocktails only after *M.bovis* challenge. Only 24 h skin-test reactions are shown for animals in this group as they had reached the humane end point. Importantly, the antigen cocktails distinguished between BCG and *M. bovis* exposure with skin test reactions occurring only in challenged animals irrespective of their immunization status (Sup. Fig. 3).

## Discussion

Bovine TB is a serious animal health problem^55^ and is one of the biggest challenges facing the cattle farming industry today^56^. It has been estimated that >50 million cattle are infected worldwide, costing an estimated US$3 billion annually^57^. Even though vaccination with *M. bovis* BCG showed widely diverging efficacies in cattle and humans^58–60^, it is still the only realistic vaccine candidate with potential benefits in reducing prevalence and spread of bovine TB in the cattle population^40,57,61^ and also reduce the severity of a herd breakdown^20,22^. The differential diagnostic tests will be required in countries where the test and slaughter control strategies are in operation^62,63^, particularly when vaccination involves BCG that will compromise the specificity of standard tuberculin-based diagnostic tools^64^. In order to control the spread of tuberculosis, effective vaccination and accurate early diagnosis of the disease are critical. Development of new and improved cattle vaccines, and associated diagnostic reagents, could contribute to improved disease control^65^.

This study is the first step in a novel strategy to engineer a diagnostically compatible BCG vaccine that has similar protective efficacy to the current commercially available BCG vaccines. We constructed a ΔBCG TK strain that gave indistinguishable protection against BTB challenge as WT BCG. We developed a compatible extended DIVA skin test that proved to be specific in not provoking skin reactions in vaccinated guinea pigs before challenge but provoking reactions post-challenge. The test, although specific, was not as sensitive as PPD-B in the Guinea pig model. Adding additional antigens in a cocktail of 6 antigens, including the prototype DIVA antigens ESAT-6, CFP-10 and Rv3615^66^, alongside our triple antigen proteins (MPB70, MPB83 and Rv3020c) led to significant increases in skin responses in all groups post-challenge whilst retaining the absence of skin test responses post-BCG vaccination prior to challenge.

We note that, as expected, the synthetic antigen cocktail did not provoke a reaction in guinea pigs inoculated with the ΔBCG TK strain, but somewhat surprisingly neither did guinea pigs inoculated with WT BCG, which still contains the antigens present in the antigen cocktail. This outcome is likely because the guinea pig study was not powered sufficiently to demonstrate the relatively small, but economically highly significant, immunoreactivity to these antigens in animals vaccinated with WT BCG. In support of this interpretation, addition of just one of these antigens, Rv3020c, to the standard DIVA cocktail of ESAT-6, CFP-10 and Rv3615c, reduced the specificity of the skin test in BCG vaccinated calves from 100% to 96% (100% specificity of ESAT-6, CFP-10, Rv3615c combination in BCG vaccinated calves in 187 tested animals vs 96% specificity ESAT-6, CFP-10, Rv3615c plus Rv3020c in 125 tested animals) (Jones and Vordermeier, unpublished observations). Although this may appear to be a relatively minor reduction in specificity, the economic and societal impact of a 4% false discovery rate when its consequence may be the culling of healthy animals is likely to prevent implementation of such a test, particularly when compared to the false-discovery rate of the standard comparative tuberculin test with of 0.02%^67^. Demonstrating a 4% false-positivity with WT BCG and loss of this false-positivity with the ΔBCG TK, advanced DIVA test combination would have required large number of guinea pigs and was beyond the scope of this project. Further, larger studies, preferentially in the cattle as target host, will be required to demonstrate this aspect of our strategy.

In summary, in this study we demonstrate, for the first time, a new strategy for engineering a live bacterial vaccine that has been rationally-designed to optimize both protection and diagnostic compatibility. The DIVA cocktail described here is specific and is not affected by vaccination. Although further experiments are needed to confirm protective efficacy and diagnostic efficacy in the bovine host, the development of combination of effective vaccine and skin test regents could transform bovine TB control programmes worldwide. Similar strategies may also be of value for control of human TB, and perhaps other infectious diseases.

## Materials and Methods

### BCG culture preparation

*M. bovis* BCG Danish 1331 (Staten’s Serum Institute, batch 111013B) was grown on Middlebrook 7H11 solid media or in Middlebrook 7H9 supplemented with 0.2% glycerol, 0.05% Tween-80 and 10% OADC at 37 °C shaking at 150 rpm in an orbital shaker^37^. When selection was required antibiotics were used at 50 µg ml^−1^ for apramycin, 50 µg ml^−1^ for hygromycin and 25 µg ml^−1^ for zeocin.

### Construction of the recombinant cosmids containing allelic exchange substrates (AESs)

Cosmid pANE001 zeomycin (Sup. Fig. 1) was derived from pYUB854 (Hyg). The original pYUB854 cosmid was modified by replacing the hygromycin resistance cassette with zeomycin cassettes. An inverse PCR of pYUB854 with primers designed to amplify plasmid backbone less the hygromycin cassette and add *Nde*1 and *Mfe*1 restriction ends^37^. The zeomycin cassettes were amplified from pNCMTB plasmid and *Nde*1 and *Mfe*1 restriction sites added to the ends^37^. The antibiotic cassette was then cloned into pYUB854, and confirmed by Sanger sequencing. The modified res–*Mfe*I-zeo–*Nde*I-res gene cassette flanked by multiple cloning sites (MCSs) was thus created.

### Construction of BCG null mutants

Mutants were generated sequentially using the mycobacteriophage-based method of specialized transduction^37,49^, and cosmids pANE001 or p0004S. Upstream (LF) and downstream (RF) sequences flanking the genes to be mutated were PCR amplified from BCG Danish genomic DNA using Qiagen High Fidelity Taq polymerase according to manufacturer’s instructions, cloned into the appropriate cosmids and confirmed by Sanger sequencing to generate the knock-out plasmids p0004S3043 (Apra), pANE3679/80 (Zeo) and pYUB2897/95 (Hyg). Primer sequences used in this study are listed in Sup. Table 4.

### Confirmation of mutant construction

As described in the earlier publication^37^, knockout genotypes were confirmed by PCR using primers outside the upstream and downstream flanking regions both alone, and in combination with antibiotic cassette specific primers (Sup. Table 4), such that PCR products would be obtained only if the antibiotic cassette was inserted in the required genomic location^50^.

### Growth analysis of BCG strains

BCG wild-type and ΔBCG TK mutant were grown to mid-log phase (OD 0.8). The cells were pelleted down and washed twice with PBS, then resuspended in 7H9 medium. This resuspended bacterium was used to inoculate fresh to a starting OD of 0.05. Growth was measured by taking OD readings. All experiments were performed in triplicate.

### *In vitro* competition assays

A mix of strains containing approximately equal amounts of the ΔBCG TK mutant and WT BCG Danish were inoculated into broth and cultured for 14 days. At selected time points the numbers of each mutant were determined by serially diluting onto selective media. Numbers of WT BCG were estimated by subtracting the antibiotic resistant colony numbers from counts from plates without antibiotics^37^. The assays were repeated three times.

### Bovine Macrophage preparations and infections

Peripheral blood mononuclear cells (PBMCs) obtained from the heparin-anticoagulated blood collected from the adult cows. The PBMCs isolated using Ficoll-Histopaque density gradient centrifugation. Monocytes from the pool of PBMCs were selected using CD14 MicroBeads (Miltenyi Biotec). The monocytes were differentiated into macrophages in 24 well plates containing complete RPMI supplemented with 1% sodium pyruvate, 1% penicillin/streptomycin and 20 ng ml^-1^ macrophage colony-stimulating factor (Miltenyi Biotec)^37^. Fresh medium was added on day 3 and infection on day 6 at a MOI of 1 with a mixed BCG culture containing approximately equal amounts of WT BCG and ΔBCG TK mutant. Control macrophages were incubated with culture medium only. After 4 h, the infected cells were washed three times with PBS. The intracellular bacilli were harvested by lysing the cells with 0.1% Triton X-100 at different time points. The mixed culture used for infection, and harvested intracellular bacilli were enumerated as described previously^37^. The assays were repeated three times.

### Cloning and expression of recombinant proteins

The fusion gene construct (GenScript, USA) of coding sequences of ESAT-6 and CFP-10, and of Rv3615c and Rv3020 of *M. tuberculosis* H37Rv were synthesized. These gene constructs were then cloned into prokaryotic expression vector pET28a (Novagen) and transformed into *E. coli* BL21 DE3 cells (Invitrogen). The protein expression was induced with 1 mM IPTG overnight at 25 °C. The His_6_ tagged ESAT-6::CFP10 and Rv3615c::Rv3020 fusion proteins were purified from the soluble fraction of the bacterial lysate using Ni–NTA agarose (immobilized metal affinity chromatography)^68^. The pooled protein fractions were dialyzed against PBS (pH 7.4) and SDS-PAGE was used to assess the purity of the protein. The protein was detected in a Western blot using anti-His_6_ antibody and the LPS from recombinant fusion proteins were removed using Triton X-100 as per the procedure^68,69^.

### Guinea pig experiments

The Guinea pig experiments were conducted according to the United Kingdom Home Office Legislation for animal experimentation and approved by a local ethical committee at Public Health England (Porton Down, United Kingdom). Dunkin Hartley guinea pigs free from pathogen-specific infection were randomly assigned to vaccine groups and identified using subcutaneously implanted microchips (Plexx, the Netherlands) to enable blinding of the analyses wherever possible^70^. Group sizes were determined by statistical power calculations (Minitab, version 16) performed using previous data (SD, approximately 0.5) to reliably detect a difference of 1.0 log_10_ in the median number of colony-forming units (cfu) per millilitre^70^. The guinea pigs were housed in groups of up to eight during vaccination and in pairs post-challenge. Animals were monitored daily for behavioural changes.

The 32 animals were divided into 4 groups (n = 8). Groups 1 and 2 were vaccinated subcutaneously on the nape with 5 × 10^4^ cfu of either ΔBCG TK (Group 1) or wild type BCG (Group 2) at day 0. Groups 3 and 4 remained unvaccinated. All groups received the pre-challenge skin tests at 34 days post-vaccination. Skin test responses (STR) were measured at 24- and 48-hours following inoculation with the antigens. Groups 1, 2 and 3 were challenged with *M. bovis* (AF2122/97) at 42 days (6 weeks) post-vaccination and received a post-challenge skin test before the scheduled cull and necropsy at 70 days (4 weeks post-challenge). Group 4 was not challenged as this was a control group to test for non-specific skin test responses. Guinea pigs in groups 1–3 were challenged by the aerosol delivery with a target estimated dose of 10–20 cfu of *M. bovis* using a contained Henderson apparatus in conjunction with an AeroMP control unit^71–73^. Fine particle aerosols of *M. bovis*, with a mean diameter of 2μm, were generated in a Collison nebulizer and delivered directly to the snout of each animal. The AeroMP is a platform system designed to manage the aerosol generation, characterization and sampling processes via a dashboard software laptop system. Throughout the study, the body weight of each animal was measured and recorded at least weekly. The frequency of checks was increased on appearance of any clinical signs or weight loss. The humane endpoint was reached when 20% loss of maximal body weight was recorded and/or observation of defined clinical signs such as laboured breathing.

The determination of bacterial load was scheduled at 4 weeks post-challenge. Guinea pigs from each group were killed and the lungs and spleens were aseptically removed and stored at −20 °C on the day of necropsy until they were processed in a single batch. On the day of tissue processing, each tissue was homogenized in 10 ml (lung) or 5 ml (spleen) sterile phosphate buffered saline (PBS). Each tissue homogenate was serially diluted in sterile PBS and 100 μl of each dilution plated, in duplicate onto Middlebrook 7H11 + OADC + pyruvate selective agar. Following incubation, colonies were enumerated to determine the colony forming units (cfu).

### Skin testing

The skin testing was performed 34 days post-vaccination prior to *M. bovis* challenge (pre-challenge skin test) and at 62 days post-vaccination around 4 weeks after *M. bovis* challenge (post-challenge skin tests). All guinea pigs, regardless of vaccination and challenge status were given PPD-B (Group A) and four specific DIVA skin test antigen preparations (Group B-E) at six separate injection sites in a Latin square formation. A diagram of the six sites for each animal (three sites on each flank) is shown in Fig. 5A. The details of the group and skin test antigen are given in Sup. Table 1.

Each antigen cocktail was prepared prior to delivery. 100 μl of each antigen preparation (2 μg of PPD-B or 1 μg of antigen cocktail preparation) was given to the appropriate site by the intradermal route. Each guinea pig received each of the five types of antigen preparation and a repeat of one other (on opposite flank) as described in Sup. Table 2. The rationale to test one preparation on both flanks of each animal was to determine whether the flank side (left or right) influenced the magnitude of the inflammatory response. We didn’t observe any significant difference in the magnitude of the skin test response when the same test was given in different locations or on either flank which nullify the influence of the position or flank of the skin test location on the magnitude of the inflammatory response (Sup. Table 3).

Skin test responses were measured at 24 h and 48 h following antigen inoculation. As we expected, the skin test reaction to recombinant proteins were lower than to PPD. Based on the observations in cattle by^33^ we defined cut-off values for positivity for the recombinant proteins at both time points at >2 mm, and >4 mm for PPD. The size of the individual erythema reactions (if present) was measured in millimetres (mm) and the average of these values was used for analysis. Skin test data were initially analysed using an ANOVA general linear model (Latin square) statistical analysis. Group comparisons of the magnitude of skin test were performed using the non-parametric Mann-Whitney test (Minitab software version 16). A test for normality was applied to the bacterial load data and the data from each vaccine group were compared and ranked using the non-parametric Mann-Whitney test (Minitab software version 16).

### Ethics approval and consent to participate

All animal experimentation was carried out within a project license granted by the United Kingdom Home Office Legislation for animal experimentation and approved by a local ethical committee at Public Health England (Porton Down, United Kingdom).

## Supplementary information

Supplementary_information

## Data Availability

Illumina and MiSeq sequence data are being submitted to the SRA database under submission SUB4615409.
