# Author Correction: Development of a diagnostic compatible BCG vaccine against Bovine tuberculosis

**DOI:** 10.1038/s41598-020-73832-4

**Published:** 2020-10-01

**Authors:** Aneesh Chandran, Kerstin Williams, Tom Mendum, Graham Stewart, Simon Clark, Sirine Zadi, Faye Lanni, Neil McLeod, Ann Williams, Bernado Villarreal-Ramos, Martin Vordermeier, Veerasamy Maroudam, Aravind Prasad, Neeraj Bharti, Ruma Banerjee, Sunitha Manjari Kasibhatla, Johnjoe McFadden

**Affiliations:** 1 School of Biosciences and Medicine, Faculty of Health and Medical Sciences, University of Surrey, Guildford, GU2 7XH UK; 2 Animal and Plant Health Agency, Addlestone, Surrey UK; 3 Public Health England, Porton Down, Salisbury, UK; 4 Translational Research Platform for Veterinary Biologicals, Chennai, India; 5 HPC-Medical and Bioinformatics Applications Group, Centre for Development of Advanced Computing, Innovation Park, Panchavati, Pashan, Pune, Maharashtra 411008 India

Correction to: *Scientific Reports* 10.1038/s41598-019-54108-y, published online 28 November 2019

Faye Lanni was omitted from the author list in the original version of this Article. This has been corrected in the PDF and HTML versions of the Article, and in the accompanying Supplementary Information file.

The Author Contributions section now reads:

A.C. generated the B.C.G. double and triple knock out, carried out the competitions experiments, data analysis and wrote the manuscript; K.W. generated libraries, individual mutants and carried out data analysis; T.M. contributed to the experiments and reviewed the manuscript; GS conceived the project and reviewed the manuscript; S.C., S.Z., N.M., F.L. & A.W. carried out the Guinea pig experiment; B.V.R. reviewed the manuscript; M.V. conceived the project and reviewed the manuscript; V.M. prepared antigen cocktail for skin test; A.P., N.B., R.B., S.M.K. carried out TRANSIT analysis; JM conceived the project and reviewed the manuscript. All authors read and approved the final manuscript.

