# Supplementary material for: Development of a diagnostic compatible BCG vaccine against Bovine tuberculosis
Source: Sci Rep. 2019 Nov 28;9:17791. doi: 10.1038/s41598-019-54108-y (PMC6882907; doi:10.1038/s41598-019-54108-y)
Supplement: Supplementary file 1 — Supplementary_information [file 41598_2019_54108_MOESM1_ESM.pdf]

Development of a diagnostic compatible BCG vaccine against Bovine tuberculosis

Aneesh Chandran[1], Kerstin Williams[1], Tom Mendum[1], Graham Stewart[1], Simon Clark[3], Sirine Zadi[3], Neil McLeod[3], Ann Williams[3], Bernardo Villarreal-Ramos[2], Martin Vordermeier[2], Veerasamy Maroudam [4], Aravind Prasad[5], Neeraj Bharti[5], Ruma Banerjee[5], Sunitha Manjari[5], Johnjoe McFadden[1†]

Supplementary Data

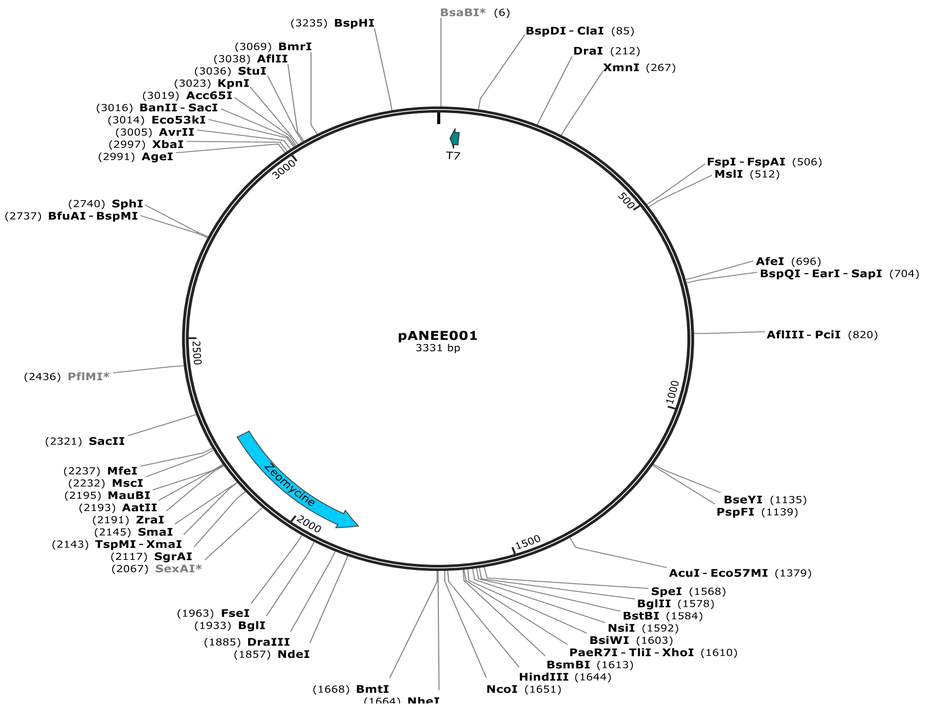

Sup. Fig 1. The cosmid map of pANEE001. The map was generated using Snap gene viewer. In this plasmid, Zeocin antibiotic cassette is flagged with MfeI and Ndel restriction sites at 3' and 5' respectively which enable the user to change the antibiotic cassette as required.

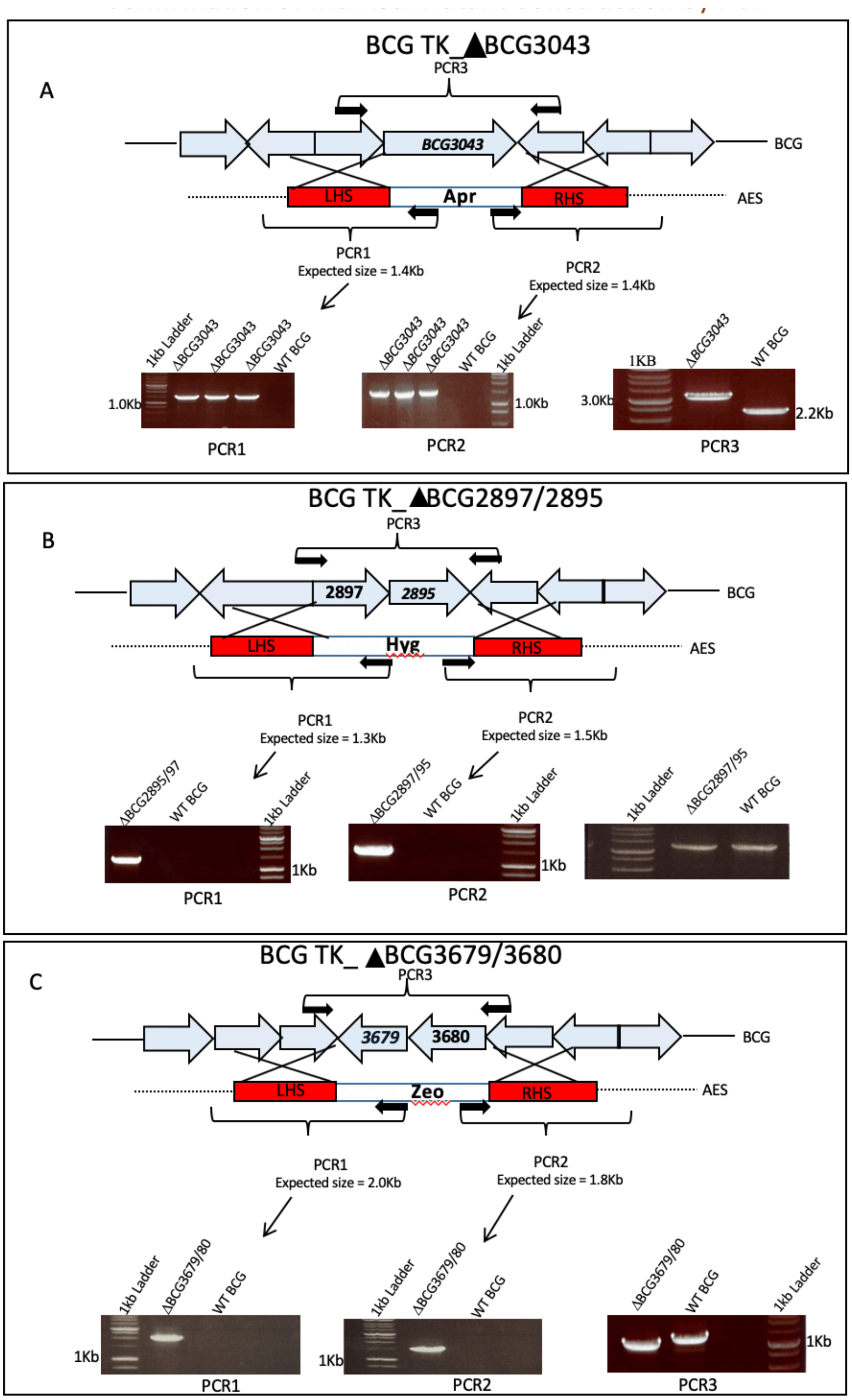

Sup. Fig 2. Confirmation of ΔBCG TK genotype by PCR. (A)PCR products of the expected size were obtained for the mutants with BCG3043_LF_CHK_R and BCG3043_LF_F (PCR1), and using BCG3043_RF_CHK_R and BCG3043_RF_R (PCR2); no products were obtained with wild type (PCRs 1 & 2). PCR over the *BCG3043* gene using BCG3043_LF_F and BCG3043_RF_R further confirms the correct insertion of the Apramycine cassette (PCR3). (B)PCR products of the expected size were obtained for the mutants with MPB70/83_LF_CHK_R and MPB70/83_LF_F (PCR1), and using MPB70/83_RF_CHK_R and MPB70/83_RF_R (PCR2); no products were obtained with wild type (PCRs 1 & 2). PCR

over the *BCG2897/95* gene using MPB70/83_LF_F and MPB70/83_RF_R further confirms the correct insertion of the Hygromycin cassette (PCR3). (C)PCR products of the expected size were obtained for the mutants with BCG3679/80_LF_CHK_R and BCG3679/80_LF_F (PCR1), and using BCG3679/80_RF_CHK_R and BCG3679/80_RF_R (PCR2); no products were obtained with wild type (PCRs 1 & 2). PCR over the *BCG3679/80* gene using BCG3679/80_LF_F and BCG3679/80_RF_R further confirms the correct insertion of the Zeocin cassette (PCR3). All PCR products were run on 1% agarose gel and imaged using a UVP gel doc machine. The schematic representations of genes are not to the scale. The empty spaces around the edges of gel images were trimmed for better representation. The untrimmed version of gel images were given below with exact same order as above images in Sup. Fig 3.

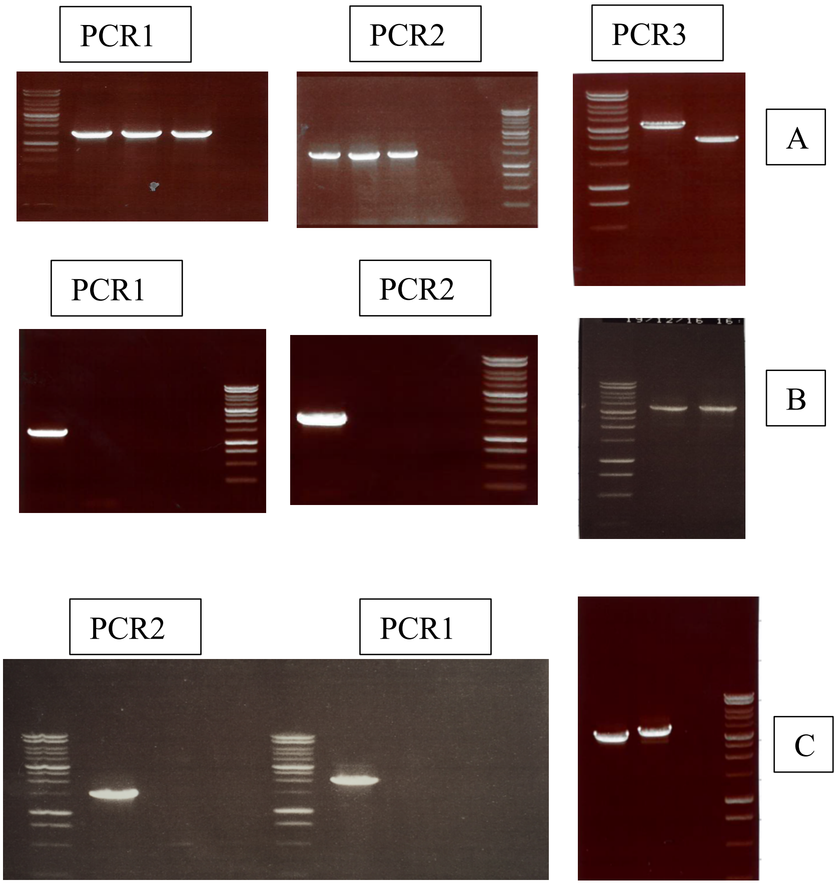

Sup. Fig 3. Confirmation of ΔBCG TK genotype by PCR. The untrimmed version of gel images of Sup. Fig 2 representing in the exact same order as Sup. Fig. 2.

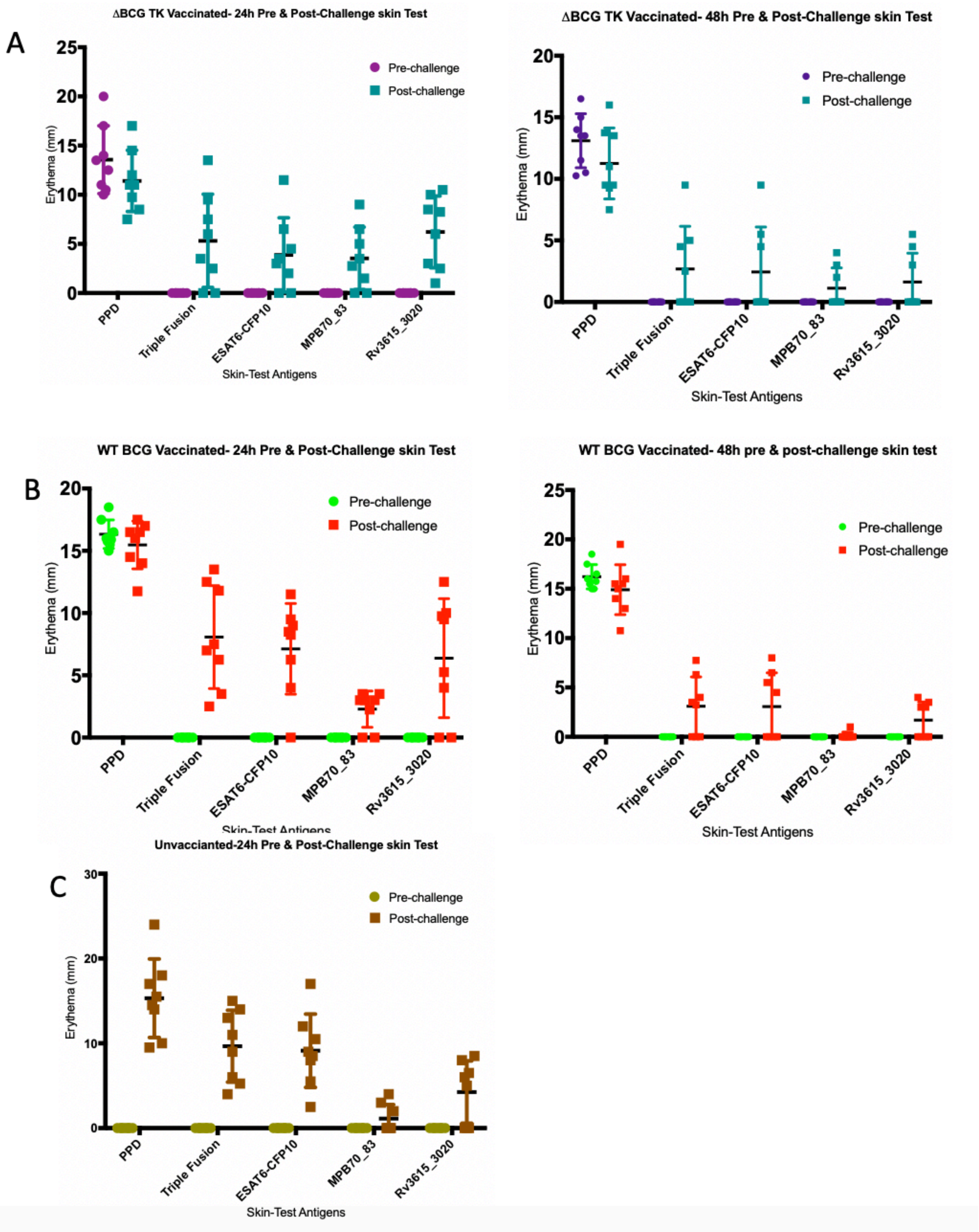

Sup. Fig 4: Pre- and Post- *M.bovis* challenge skin test at 24h and 48h against immunodominant antigens. (A, B) The size of erythema against PPD and Fusion antigens in the vaccinate guinea pigs, pre and post *M. bovis* challenge. (C) The size of erythema against PPD and Fusion antigens in the unvaccinated guinea pigs, pre and post *M. bovis* challenge. The error bars indicate standard deviation for each vaccine group.

Skin Test Group	Antigens
A	PPD-B
B3 (Triple Fusion)	ESAT6-CFP10 Fusion
	MPB70-MPB83 Fusion
	Rv3615c-Rv3020c Fusion
C	ESAT6-CFP10 Fusion
	PBS
D	MPB70-MPB83 Fusion
E3	RV3615c-RV3020c Fusion

Sup.Table1: Details of the skin test antigens. The table list the antigen components for each skin test group

		right flank			left flank		
		Injection Sites					
Vaccine	Guinea Pig ID	1	2	3	4	5	6
TK/ Vaccine Group 1 (ΔMPB70/83_ ΔRv3615/16_ ΔRv3020)	53587	A	B3	C	D	E3	A
	46349	B3	C	D	E3	A	B3
	22385	C	D	E3	A	B3	C
	53970	D	E3	A	B3	C	D
	73412	E3	A	B3	C	D	E3
	73656	A	B3	C	D	E3	A
	53286	B3	C	D	E3	A	B3
	46461	C	D	E3	A	B3	C

		right flank					
		Injection Sites					
Vaccine	Guinea Pig ID	1	2	3	4	5	6
WT Danish Control/ Vaccine Group 2 (no knockouts)	22603	A	B3	C	D	E3	A
	27113	B3	C	D	E3	A	B3
	46267	C	D	E3	A	B3	C
	53393	C	D	E3	A	B3	C
	73404	C	A	B3	C	D	E3
	22504	A	B3	C	D	E3	A
	53775	B3	C	D	E3	A	B3
	53446	C	D	E3	A	B3	C

		right flank			left flank		
		Injection Sites					
Vaccine	Guinea Pig ID	1	2	3	4	5	6
Unvaccinated & Challenged Group 3	23146	A	B3	C	D	E3	A
	46256	B3	C	D	E3	A	B3
	74064	C	D	E3	A	B3	C
	66922	D	E3	A	B3	C	D
	53568	E3	A	B3	C	D	E3
	73358	A	B3	C	D	E3	A
	26639	B3	C	D	E3	A	B3
	46990	C	D	E3	A	B3	C

		right flank			left flank		
		Injection Sites					
Vaccine	Guinea Pig ID	1	2	3	4	5	6
Unvaccinated & Unchallenged Group 4	46568	A	B3	C	D	E3	A
	73776	B3	C	D	E3	A	B3
	46761	C	D	E3	A	B3	C
	23009	D	E3	A	B3	C	D
	46343	E3	A	B3	C	D	E3
	46999	A	B3	C	D	E3	A
	46428	B3	C	D	E3	A	B3
	44387	C	D	E3	A	B3	C

Sup. Table2: Pre-challenge & post-challenge skin testing study design (Skin-testing injection Regime). Table shows the injection for each antigen preparations (coded A-E) for each group of animals. Antigen preparation group Key- A: PPD-B, B3: ESAT6-CFP10 fusion+MPB7—83 fusion+Rv3615c-3020 fusion(Triple fusion), C:ESAT6-CFP10 fusion, D: MPB70—83 fusion, E3:RV3615c-Rv3020c fusion

Latin Square preliminary analysis			
24 hour TST reading		General Linear model ANOVA (P value)	
		Pre-Challenge	Post-Challenge
Vaccine	Treatment	Position on flank: signal of antigen	Position on flank: signal of antigen
TK	ΔBCG TK	0.792	0.323
WT BCG	WT BCG strain	0.339	0.889
Unvaccinated + challenged	Unvaccinated	#	0.362
Unvaccinated + unchallenged	Unvaccinated	#	0.516

Latin Square preliminary analysis			
48 hour TST reading		General Linear model ANOVA (P value)	
		Pre-Challenge	Post-Challenge
Vaccine	Treatment	Position on flank: signal of antigen	Position on flank: signal of antigen
TK	ΔBCG TK	0.762	0.072
WT BCG	WT BCG strain	0.642	0.074
Unvaccinated + challenged	Unvaccinated	#	NA
Unvaccinated + unchallenged	Unvaccinated	#	0.451

Sup. Table 3: ANOVA general linear model (Latin square) statistical analysis to determine the effect of skin test flank location. The # symbol denotes the statistical analysis could not generate P value as all responses were 0. NA denotes not done

Zeo_casset_F	GAACTCCAATTGATGGCCAAGTTGACCAGTGC
Zeo_casset_R	GAACTCCATATGTCAGTCCTGCTCCTCGGCCAC
pYUB_inv_F	GACATC CAATTGTCACAGCGGACCTCTATTC
pYUB_inv_R	GATCTCCATATGAACTGGCGCAGTTCCTCTGG
BCG3043_RF_F	GATCTCAAGCTTTCCTTCCAATTCGAATC
BCG3043_RF_R	GATCTCACTAGTTGGTGGCGACGAATTTC
BCG3043_LF_F	GATCTCCTTAAGCCAACCACGCCACATAC
BCG3043_LF_R	GATCTCTCTAGATGCTCGGAATGAAAAGG
MPB70/83_RF_F	GATCTCAAGCTTATGCCTCCGGCGTAATC
MPB70/3_RF_R	GATCTCACTAGTGAGCCCTGACCATTTCC
MPB70/83_LF_F	GATCTCCTTAAGGCTCGTCAGCGACGGC
MPB70/83_LF_R	GATCTCTCTAGAACCAGTGATTCGGAGTG
BCG3679/80_RF_F	GATCTCAAGCTTCCTGACCACGTTTGCTGC
BCG3679/80_RF_R	GATCTCACTAGTCGTGCTCTATTAATGCTG
BCG3679/80_LF_F	GATCTCCTTAAGTCTATCAGTAGGCGGCTAG
BCG3645/46_LF_R	GATCTCTCTAGAAACTGCGCTGCGACAATG
BCG3043_RF_CHK_F	GTCGTTGCAGAGTGCGGTGG
BCG3043_LF_CHK_R	CCAATAATGTTGAAACCCAGG

MPB70/83_RF_CHK_F	CCAGCGATTCCTTGTTG
MPB70/83_LF_CHK_R	CAAAACACGAACAAGTGAGG
BCG3679/80_RF_ CHK_ F	AAATCGCGTACGTGG
BCG3679/80_ RF_CHK_R	GAAGTGCACGCAGTTGCC
BCG3679/80_LF_CHK_ F	CAAGTTGACCAGTGCCGTTC
BCG3679/80_LF_CHK_ R	CAATTGAGTCATCCAGCG

Sup. Table4: Primers used in the study
